# Independent validation of machine learning in diagnosing breast Cancer on magnetic resonance imaging within a single institution

**DOI:** 10.1186/s40644-019-0252-2

**Published:** 2019-09-18

**Authors:** Yu Ji, Hui Li, Alexandra V. Edwards, John Papaioannou, Wenjuan Ma, Peifang Liu, Maryellen L. Giger

**Affiliations:** 1 Department of Breast Imaging, National Clinical Research Center for Cancer, Tianjin Medical University Cancer Institute and Hospital, Tianjin, China; 2 Key Laboratory of Cancer Prevention and Therapy; Tianjin’s Clinical Research Center for Cancer; Key Laboratory of Breast Cancer Prevention and Therapy, Ministry of Education, Tianjin Medical University, Tianjin, 30060 China; 3 Department of Radiology, University of Chicago, 5841 S Maryland Ave, MC2026, Chicago, IL 60637 USA

**Keywords:** Computer-aided diagnosis, Breast cancer, Quantitative MRI, Radiomics, Machine learning, Artificial intelligence (AI), Independent statistical testing

## Abstract

**Background:**

As artificial intelligence methods for the diagnosis of disease advance, we aimed to evaluate machine learning in the predictive task of distinguishing between malignant and benign breast lesions on an independent clinical magnetic resonance imaging (MRI) dataset within a single institution for subsequent use as a computer aid for radiologists.

**Methods:**

Computer analysis was conducted on consecutive dynamic contrast-enhanced MRI (DCE-MRI) studies from 1483 breast cancer and 496 benign patients who underwent MRI examinations between February 2015 and October 2017; with the age ranges of the cancer and benign patients being 19 to 77 and 16 to 76 years old, respectively. Cases were separated into a training dataset (years 2015 & 2016; 1444 cases) and an independent testing dataset (year 2017; 535 cases) based solely on MRI examination date. After radiologist indication of the lesion, the computer automatically segmented and extracted radiomic features, which were subsequently merged with a support-vector machine (SVM) to yield a lesion signature. Area under the receiving operating characteristic (ROC) curve (AUC) with 95% confidence intervals (CI) served as the primary figure of merit in the statistical evaluation for this clinical classification task.

**Results:**

In the task of distinguishing malignant and benign breast lesions DCE-MRI, the trained predictive model yielded an AUC value of 0.89 (95% CI: 0.858, 0.922) on the independent image set. AUC values of 0.88 (95% CI: 0.845, 0.926) and 0.90 (95% CI: 0.837, 0.940) were obtained for mass lesions only and non-mass lesions only, respectively. Compared with actual clinical management decisions, the predictive model achieved 99.5% sensitivity with 9.6% fewer recommended biopsies.

**Conclusion:**

On an independent, consecutive clinical dataset within a single institution, a trained machine learning system yielded promising performance in distinguishing between malignant and benign breast lesions.

## Background

Breast cancer is the most common cancer and the second leading cause of cancer death in women in western countries [1]. In Chinese women, breast cancer is the most common cancer diagnosed, and it alone is expected to account for 15% of all new cancers in women [2]. Dynamic contrast enhanced (DCE) magnetic resonance imaging (MRI) of the breast is being used increasingly for a variety of clinical purposes, including screening of women at high risk for developing breast cancer, evaluating of the extent of malignant disease, and post-treatment evaluation [3–5]. DCE-MRI has emerged as a modality that is complementary to mammography and ultrasonography because of the additional three-dimensional spatial and temporal information about the lesion that it yields.

While there is diagnostic value of DCE-MRI characterization in the differentiation of malignant from benign lesions [6], the MRI assessment of breast cancer cases may be hindered by inter-observer and intra-observer variations, labor-intensive interpretation methods, and limited clinical interpretation guidelines [7, 8]. To aid radiologists in diagnostic classification, various investigators are developing computerized image analysis methods for characterization, i.e., computer-aided diagnosis (CADx)/radiomics [9–15]. The purpose of this study was to evaluate the potential of quantitative MRI radiomics and machine learning in the task of distinguishing between malignant and benign breast lesions on an independent, consecutive clinical dataset within a single institution for ultimate use as a computer aid to radiologists in the workup of breast lesions. To our knowledge, our study is the largest such independent study in the field.

## Methods

### Breast DCE-MRI database

**Fig. 1:**
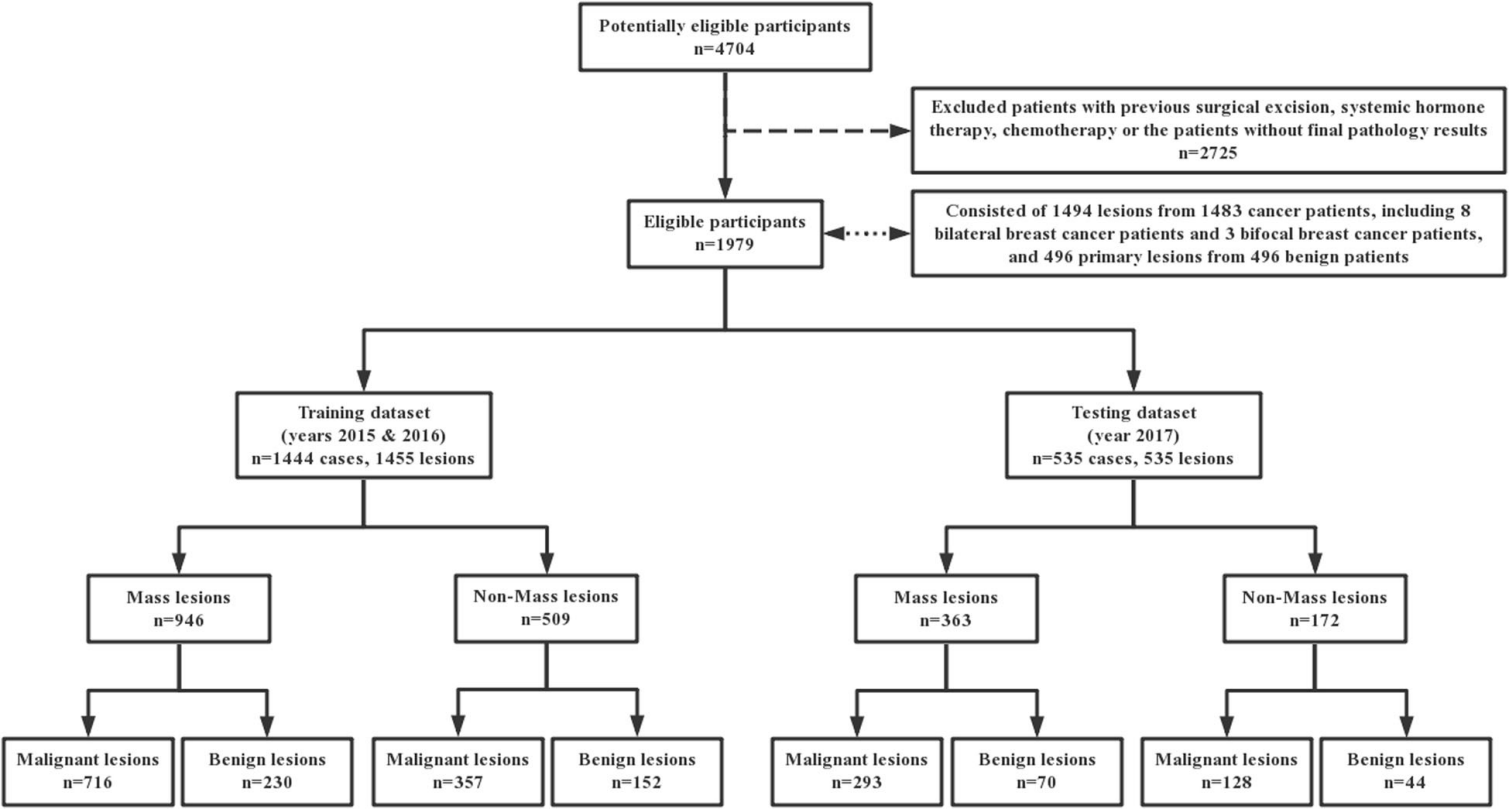
Flowchart of study participants enrollment

Our study initially involved 4704 patients presenting for breast DCE-MRI examinations as recorded in the Department of Breast Imaging of the Tianjin Medical University Cancer Institute and Hospital. As this study was a retrospective and anonymized machine learning study, informed consent was waived and the study was deemed exempt. Patient’s MRIs and clinical data were collected consecutively for our study within the years of 2015–2017. Exclusion criteria included patients with either previous surgical excision, systemic hormone therapy, chemotherapy or the patients without final pathology results. A total of 1979 patients were ultimately included in our study (Fig. 1).

We conducted a retrospective review of the breast MRI images from the 1483 histopathology-proven breast cancer patients and the 496 histopathology-proven benign patients who had underwent diagnostic breast MRI examinations between February 2015 and October 2017. All histopathology was based on surgical specimens. The age range of the cancer patients was between 19 and 77 years old with an average of 48.1 years with a standard deviation of 9.9 years and a median of 47 years. The age range of the benign patients was between 16 and 76 years old with an average of 42.1 years with a standard deviation of 9.8 years and a median of 43 years. The breast MRI databases consisted of 1494 lesions from the 1483 cancer patients, including 8 bilateral breast cancer patients and 3 bifocal breast cancer patients, and 496 primary lesions from 496 benign patients.

MR images had been obtained with a 3 T GE system using a dedicated 8-channel phased-array breast coil (Discovery 750, GE Medical Systems, Milwaukee, WI). Sagittal dynamic contrast-enhanced MRI (DCE-MRI) was obtained with the volume imaging for breast assessment (VIBRANT) bilateral breast imaging technique, with TR = 6.1 ms, TE = 2.9 ms, flip angle = 15°, matrix size = 256 × 128, field of view = 26 cm × 26 cm, NEX = 1, slice thickness = 1.8 mm. The temporal resolution for each dynamic acquisition was 90 s. Before injection of the contrast agent, serial mask images were obtained. Successively, the contrast agent (Gd-DTPA, 0.1 mmol/kg body weight, flow rate 2.0 ml/s) was injected using an automatic MR-compatible power injector, and followed by flushing with the same total dose of saline solution. Dynamic MRI acquisitions were started immediately after the injection. The acquisition was repeated five times, and each phase took 90 s.

In order to not incur bias in case selection as well as to mimic a development-then-clinical-use scenario, our database was divided into a training dataset and a testing dataset based solely on the date of the MRI examinations. The training data set included the breast MRIs acquired within February 2015 through December 2016, and the test dataset included the breast MRIs acquired within January 2017 through October 2017. Note that the cases were unique in that no patients were within both the training and testing sets.

**Fig. 2:**
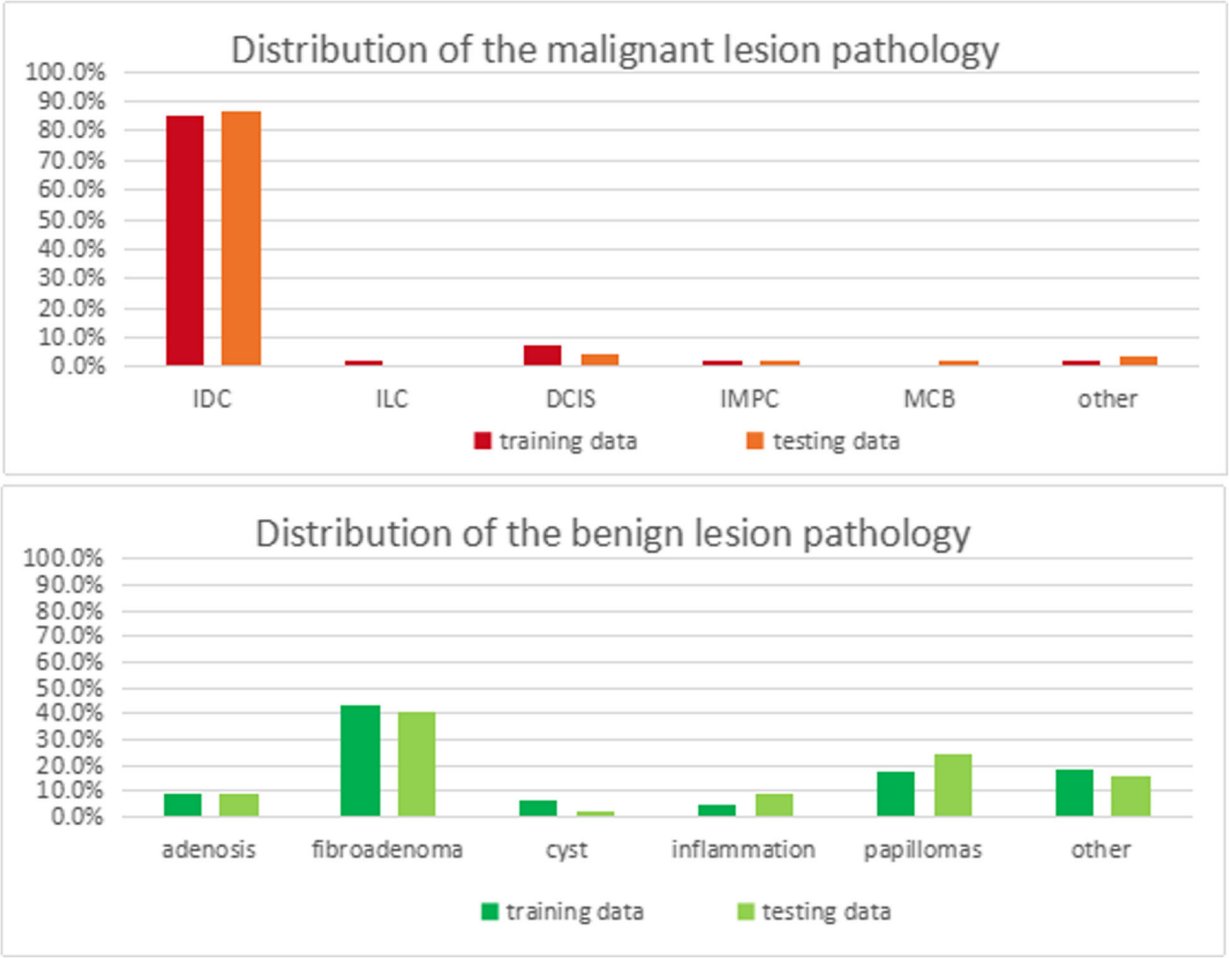
Distribution of unique patients relative to their primary lesion pathology (malignant and benign) in the training and testing data sets. IDC: invasive ductal carcinoma; ILC: infiltrating lobular carcinoma; DCIS: ductal carcinoma in situ; IMPC: invasive micropapillary carcinoma; MCB: mucinous carcinoma of the breast

The clinicopathological characteristics of the breast cancer and benign patients of the two datasets are shown in Table 1, including the BI-RADS classifications. Invasive ductal carcinomas composed the majority of malignant lesions, whereas fibroadenomas were the most common benign lesion (Fig. 2). During the patients’ clinical workup, BI-RADS ratings had been recorded by the MRI radiologist using the Breast Imaging Reporting and Data System (BI-RADS) [16]. Note that all of the patients in this study underwent pathological examination, even those with MRI-BI-RADS categories 1 or 2 or 3 when their mammographic or their sonographic findings were judged to be suspicious or highly suggestive for cancer, and the actual clinical decisions were made according to the multimodality medical imaging interpretations.

**Table 1:** Clinicopathological characteristics of breast cancer and benign patients

Clinicopathological characteristics of breast cancer and benign patients				
	Training data		Testing data	
	Malignant	Benign	Malignant	Benign
Total	1073	382	421	114
Age, years (mean, range)	47.6 (19–77)	42.2 (16–76)	49.3 (25–75)	41.9 (19–65)
Lesion type				
Mass	716 (66.7%)	230 (60.2%)	293 (69.6%)	70 (61.4%)
Non-mass	357 (33.3%)	152 (39.8%)	128 (30.4%)	44 (38.6%)
MRI-BI-RADS category				
0	0 (0%)	2 (0.5%)	0 (0%)	0 (0%)
1	0 (0%)	1 (0.3%)	0 (0%)	2 (1.8%)
2	0 (0%)	4 (1.0%)	0 (0%)	0 (0%)
3	4 (0.3%)	202 (52.9%)	0 (0%)	50 (43.8%)
4	351 (33.1%)	170 (44.5%)	113 (26.8%)	60 (52.6%)
5	529 (49.8%)	3 (0.8%)	221 (52.5%)	2 (1.8%)
6	178 (16.8%)	0 (0%)	87 (20.7%)	0 (0%)
Pre or Post Biopsy MRI				
Pre	868 (81.7%)	362 (94.8%)	330 (78.4%)	112 (98.2%)
Post	194 (18.3%)	20 (5.2%)	91 (21.6%)	2 (1.8%)
Histology				
IDC	914 (85.2%)		366 (86.9%)	
ILC	22 (2.1%)		4 (1.0%)	
DCIS	76 (7.1%)		18 (4.3%)	
Other malignant lesions	61 (5.6%)		33 (7.8%)	
Fibroadenoma		165 (43.2%)		46 (40.4%)
Papilloma		66 (17.3%)		28 (24.6%)
Inflammation		19 (5.0%)		10 (8.8%)
Other benign lesions		132 (34.5%)		30 (26.3%)
Grade of IDC				
I	56 (6.2%)		13 (3.7%)	
II	683 (75.1%)		275 (77.2%)	
III	171 (18.7%)		68 (19.1%)	
Lymph node status (*n* = 1468)				
Negative	734 (70.3%)		295 (70.7%)	
Positive	310 (29.7%)		122 (29.3%)	
Estrogen receptor				
< 1%	193 (18.1%)		77 (18.3%)	
≥ 1%	876 (81.9%)		344 (81.7%)	
Progesterone receptor				
< 1%	222 (20.8%)		104 (24.7%)	
≥ 1%	846 (79.2%)		317 (75.3%)	
Her2				
0 or 1+	632 (59.2%)		243 (57.7%)	
2+ or 3+	436 (40.8%)		178 (42.3%)	
Ki-67				
< 14%	180 (16.9%)		60 (14.3%)	
≥ 14%	887 (83.1%)		361 (85.7%)

### Computerized analysis of breast lesions on MRI images

**Fig. 3:**
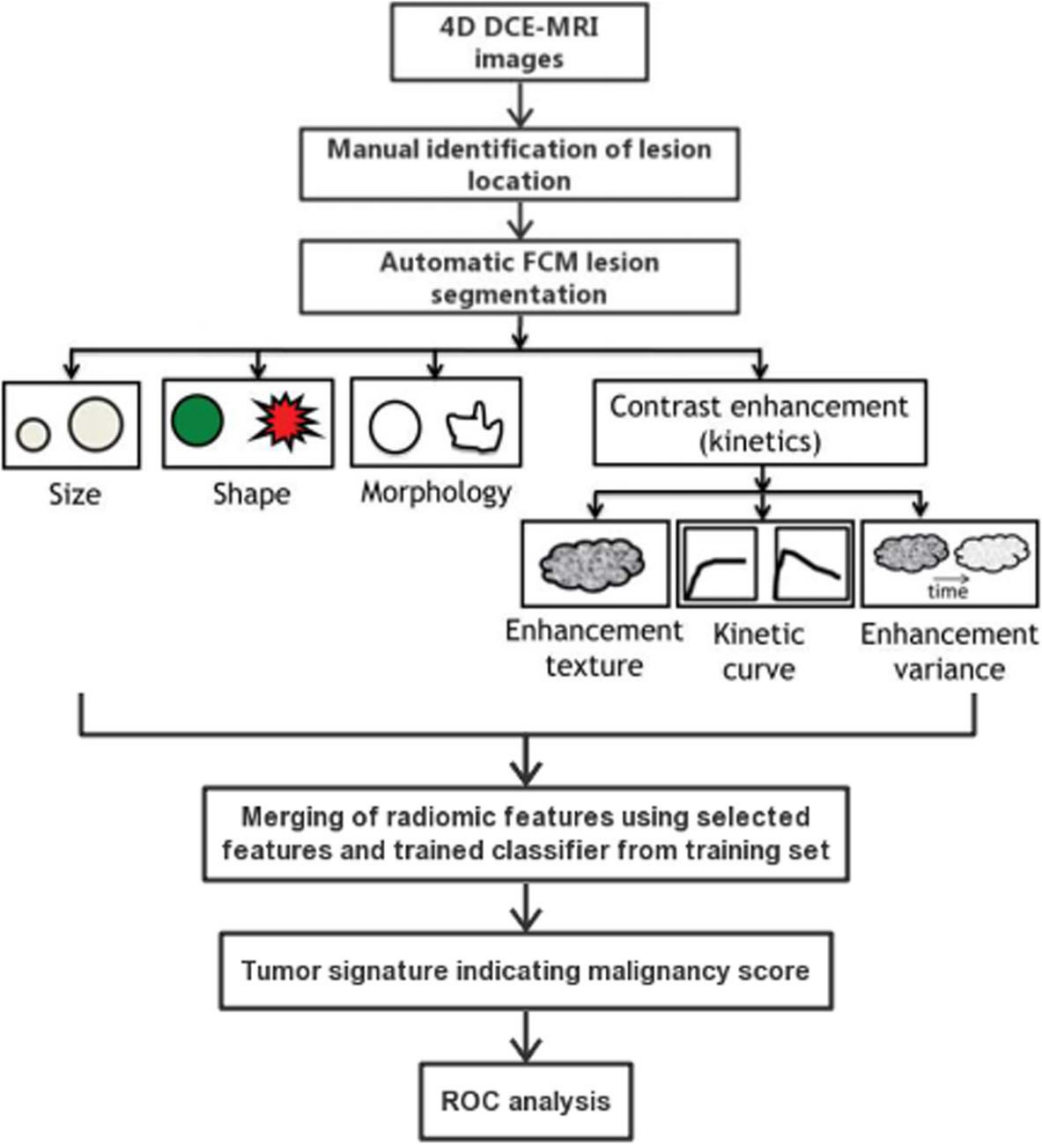
Diagram outlines the protocol for automated analysis of breast lesions seen on DCE MR imaging

We analyzed the DCE-MRIs using an existing quantitative radiomics machine learning workstation from the University of Chicago, which had been previously developed to characterize suspicious breast lesions on MRI as benign or malignant (Fig. 3) [11, 17–19]. With the workstation, a breast lesion is first manually located on the MRI by the study radiologist (YJ), a breast radiologist with 5 years of experience in breast DCE-MRIs. The computer then automatically conducted three-dimensional segmentation of the tumor and extraction of radiomic features, including those from six categories: size, shape, morphology, enhancement texture, kinetics, and enhancement-variance kinetics.

The output from this established workstation was subsequently used for the machine learning predictive model to perform classification—that is, calculation of a malignancy score related to the likelihood of malignancy for each lesion.

During training of the predictive model on the training set, stepwise feature selection using linear discriminant analysis with a Wilks lambda cost function [20] was conducted in order to identify the subset of features that performed effectively in the classification of malignant and benign lesions [21]. Then a support-vector machine (SVM) classifier [22] was trained yielding a lesion score, related to the likelihood of malignancy.

The diagnostic performance was evaluated using the trained predictive model on the independent test set – for (a) all cases, both mass and non-mass lesions, (b) only mass lesions, and (c) only non-mass lesions. In order to assess the robustness of the trained system, only the one trained system was used in all three evaluations. Such evaluations were deemed to mimic the clinical situation where the mass/non-mass status of a lesion is unknown.

### Performance evaluation and statistical analyses

Receiver operating characteristic (ROC) analysis was used to assess overall classification performance on the independent test set for the task of differentiating between malignant and benign lesions: (a) for all lesions; i.e., both mass and non-mass lesions, (b) only mass lesions, and (c) only non-mass lesions. Area under the ROC curve (AUC) served as the primary figure of merit in these tasks [23, 24]. Secondary performance metrics calculated were sensitivity, specificity, positive predictive value (PPV) and negative predictive value (NPV) [25].

Note that the BI-RADS had been used by the radiologist during the actual clinical interpretation in which all available MR images were used. And although BI-RADS categories 1, 2 and 3 are considered benign and categories 4 and 5 are considered malignant, clinically, all lesions had been sent to biopsy. Therefore, the clinical performance could be characterized as having 100% sensitivity and 0% specificity.

Thus, for comparison of the machine learning system to the actual clinical findings, the threshold value of the computer-generated malignancy score that resulted in 100% sensitivity on the training set was determined and subsequently applied to the testing set to obtain sensitivity, specificity, PPV, and NPV values. Resulting performance values at different threshold values were also calculated. PPV is calculated as the percentage of true positives over all lesions that had been classified as positive (i.e., malignant) by the trained predictive model, i.e., the probability that a case with a malignant computer output actually has cancer. NPV is the percentage of true negatives over all lesions that had been classified as negative (i.e., benign) by the trained predictive model, i.e., the likelihood that a case with a benign computer output actually is cancer free.

All statistical analyses were performed using SPSS software (version 19.0, SPSS). The reported *p*-values were two-sided. A *p*-value less than 0.05 was set as the threshold for statistical significance given that a single performance evaluation was conducted. In addition, confidence intervals were calculated using ROC software.

## Results

Radiomic features, which had been selected and merged into the lesion signature during training included 2 shape phenotypes, 1 morphological phenotype, 3 enhancement texture phenotypes, and 4 kinetic curve assessments (Table 2).

**Table 2:** Summary of computerized features in distinguishing between malignant and benign on dynamic contrast-enhanced magnetic resonance imaging.

Feature	Description
Irregularity	Deviation of the lesion surface from the surface of a sphere
Surface to volume ratio (1/mm)	Ration of surface area to volume
Margin sharpness	Mean of the image gradient at the lesion margin
Energy	Measure of image homogeneity
Information measure of correlation	Measure of nonlinear gray-level dependence
Sum Average	Measure of the overall image brightness
Maximum enhancement	Maximum contrast enhancement
Time to peak	Time at which the maximum enhancement occurs
Washout rate (1/s)	Washout speed of the contrast enhancement
Volume of most enhancing voxels (mm^3^)	Volume of the most enhancing voxels

**Fig. 4:**
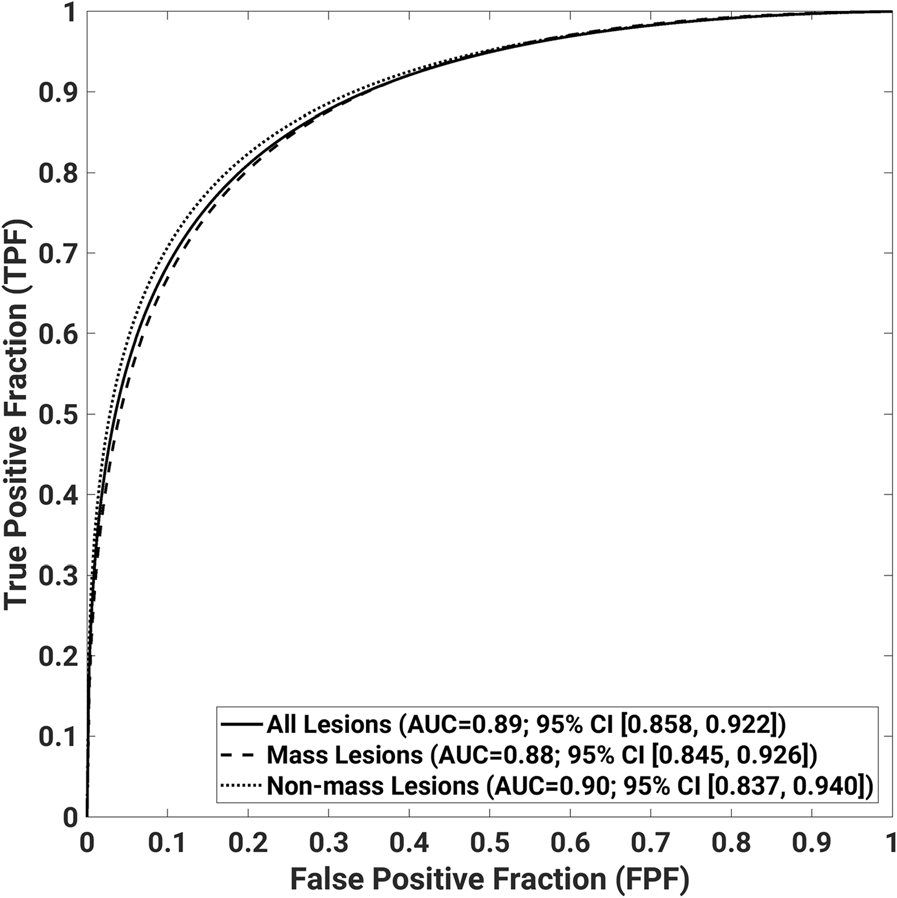
Receiver operating characteristic curves for the classification performance of the trained radiomics signature on the independent clinical testing set for (**a**) malignant and benign lesions, (**b**) malignant and benign mass lesions, (**c**) malignant and benign non-mass lesions

On the independent test dataset including both mass and non-mass lesions, the trained machine learning system yielded an AUC value of 0.89 (95% CI: 0.858, 0.922) in the task of distinguishing between malignant and benign mass lesions (Fig. 4).

For mass lesions in the test dataset, the trained system yielded an AUC value of 0.88 (95% CI: 0.845, 0.926). For non-mass lesions in the test dataset, the trained system yielded an AUC value of 0.90 (95% CI: 0.837, 0.940).

Summary of sensitivity, specificity, PPV, and NPV values at different threshold values of the malignancy score in test set are given in Table 3. At the threshold value that had yielded 100% sensitivity on the training set, the machine learning system on both mass and non-mass lesions demonstrated on the test set a higher PPV (i.e., 80.3%, 419/522) than the actual clinical decisions (78.7%, 421/535) (*P* > 0 .05), that is, it suggested eleven fewer unnecessary benign biopsies (i.e., 9.6%, 11/114). However, it erroneously would have not recommended biopsy of two cancers (i.e., 0.5%, 2/421). These two cases were both invasive ductal carcinomas and were initially classified by the radiologist as BI-RADS 5, and thus, would have gone to biopsy.

**Table 3:** Summary of sensitivity, specificity, PPV and NPV at different threshold values of the malignancy score on the independent test set

	Performance on All Lesions in Test Set				Performance on Mass Lesions in Test Set				Performance on Non-mass enhancements on Test Set			
Malignancy score threshold	Sensitivity	Specificity	PPV	NPV	Sensitivity	Specificity	PPV	NPV	Sensitivity	Specificity	PPV	NPV
0.00756 [Threshold value yielding 100% sensitivity on the training set]	99.5%, 419/421	9.6%, 11/114	80.3%, 419/522	84.6%, 11/13	99.3%, 291/293	10.0%, 7/70	82.2%, 291/354	77.8%, 7/9	100.0%, 128/128	9.1%, 4/44	76.2%, 128/168	100.0%, 4/4
0.05	98.1%, 413/421	35.1%, 40/114	84.8%, 413/487	83.3%, 40/48	97.6%, 286/293	38.6%, 27/70	86.9%, 286/329	79.4%, 27/34	99.2%, 127/128	29.5%, 13/44	80.4%, 127/158	92.9%, 13/14
0.1	96.7%, 407/421	43.0%, 49/114	86.2%, 407/472	77.8%, 49/63	96.6%, 283/293	47.1%, 33/70	88.4%, 283/320	76.7%, 33/43	96.9%, 124/128	36.4%, 16/44	81.6%, 124/152	80.0%, 16/20
0.2	94.3%, 397/421	54.4%, 62/114	88.4%, 397/449	72.1%, 62/86	94.9%, 278/293	55.7%, 39/70	90.0%, 278/309	72.2%, 39/54	93.0%, 119/128	52.3%, 23/44	85.0%, 119/140	71.9%, 23/32
0.3	91.9%, 387/421	64.9%, 74/114	90.6%, 387/427	68.5%, 74/108	93.2%, 273/293	65.7%, 46/70	91.9%, 273/297	69.7%, 46/66	89.1%, 114/128	63.6%, 28/44	87.7%, 114/130	66.7%, 28/42
0.4	87.9%, 370/421	73.7%, 84/114	92.5%, 370/400	62.2%, 84/135	88.7%, 260/293	74.3%, 52/70	93.5%, 260/278	61.2%, 52/85	85.9%, 110/128	72.7%, 32/44	90.2%, 110/122	64.0%, 32/50
0.5	83.6%, 352/421	82.5%, 94/114	94.6%, 352/372	57.7%, 94/163	83.3%, 244/293	84.3%, 59/70	95.7%, 244/255	54.6%, 59/108	84.4%, 108/128	79.5%, 35/44	92.3%, 108/117	63.6%, 35/55
0.6	75.8%, 319/421	89.5%, 102/114	96.4%, 319/331	50.0%, 102/204	75.1%, 220/293	88.6%, 62/70	96.5%, 220/228	45.9%, 62/135	77.3%, 99/128	90.9%, 40/44	96.1%, 99/103	58.0%, 40/69
0.7	61.3%, 258/421	91.2%, 104/114	96.3%, 258/268	39.0%, 104/267	60.1%, 176/293	91.4%, 64/70	96.7%, 176/182	35.4%, 64/181	64.1%, 82/128	90.9%, 40/44	95.3%, 82/86	46.5%, 40/86
0.8	46.1%, 194/421	96.5%, 110/114	98.0%, 194/198	32.6%, 110/337	43.0%, 126/293	97.1%, 68/70	98.4%, 126/128	28.9%, 68/235	53.1%, 68/128	95.5%, 42/44	97.1%, 68/70	41.2%, 42/102
0.9	20.0%, 84/421	99.1%, 113/114	98.8%, 84/85	25.1%, 113/450	17.7%, 52/293	98.6%, 69/70	98.1%, 52/53	22.3%, 69/310	25.0%, 32/128	100.0%, 44/44	100.0%, 32/32	31.4%, 44/140

Compared with non-mass lesions, the machine learning system demonstrated a lower sensitivity (*P* > 0.05) and higher specificity on mass lesions (*P* > 0.05).

**Fig. 5:**
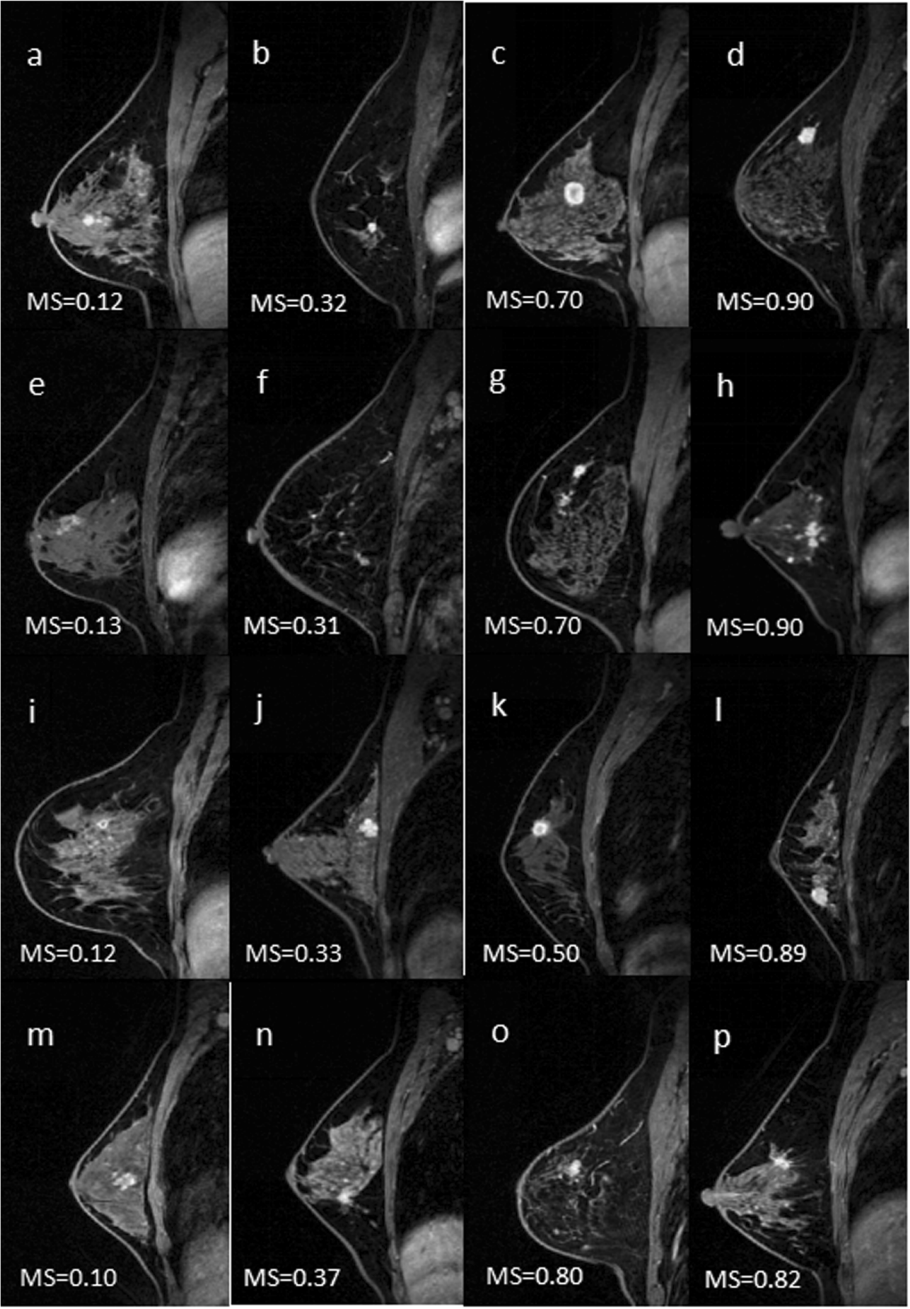
Some representative breast MRI studies from the independent consecutive test set as classified by the trained MRI radiomic signature. (a-d) Malignant mass examples; (e-h) Malignant non-mass examples; (i-l) Benign mass examples; (m-p) Benign non-mass examples

Some representative breast DCE-MRI studies from the independent consecutive test set as classified by the trained MRI machine learning system are presented in Fig. 5.

## Discussion

Our results demonstrated that a computer workstation, initially developed with datasets from the US for automatic 3D lesion segmentation and radiomic feature extraction, has the potential to distinguish between malignant and benign breast lesions from Chinese populations. It is important to note that the statistical power of the current study was limited by the modest size of the database, even though, to our knowledge, this is the largest database of this type in this breast radiomics field. Our results demonstrate that machine learning analysis of DCE-MRI may potentially provide clinically-useful information to distinguish benign and malignant lesions in Chinese databases obtained from a single institution.

While we cannot compare directly to the reported results from others due to the use of different databases, we can note that the performance level of the computer workstation was similar, and often higher, than other reported AUCs in this diagnostic task [26–28]. We also note that our performance was higher than that reported in Shimauchi et al. [29], which indicated that use of the computer aid resulted in a statistically significant improvement in radiologists’ performances.

The American College of Radiology (ACR) BI-RADS MRI lexicon [16] is used worldwide for describing the morphologic and kinetic features of breast lesions. It allows for standardization of the terminology used in describing the findings and categorization of the study. Subsequent descriptors of other lesion features, such as shape, distribution, margins, enhancement pattern are also used, which differ depending on the type of enhancement, i.e., mass enhancement or non-mass enhancement. Most previous investigations have reported on masses and rarely for lesions presenting as non-mass enhancement, primarily because of the challenges in defining the lesion extent for computer-based analysis. In our study, in order to mimic clinical practice, a single and independently-trained machine learning model was used for all the lesion types (masses and non-mass enhancements), and our result demonstrated that the classification model was stable in the task of distinguishing between malignant and benign for mass and non-mass lesions.

Note that in clinical practice, radiologists’ performance is based on multiparametric breast MR images, including DCE, T2-weighted, and diffusion-weighted images, as well as mammography and ultrasound. In our study, the computer only analyzed dynamic contrast-enhanced MR images to yield the predictive lesion signature. One would expect improved performance by using multiparametric breast MR images and multimodality medical images; thus, we will analyze those in the future.

The imaging technique used in our study involves acquisition of one pre-contrast and a series of post-contrast images of both breasts at a temporal resolution of roughly 90 s. This type of breast MRI acquisition sequence has the advantage of being able to provide both morphological and kinetic information from one MRI examination, and was representative of early dynamic MRI protocols [30]. In addition, our large clinical database came from a single institution, thus, handling the problem that the image acquisition protocols across breast MRIs might not be standardized. However, that also limits statements on generalizability of the findings.

Patient motion during image acquisition may introduce inaccuracies in the computer-extracted kinetic features [31, 32]. Cases with abrupt and large patient movements between dynamic series had been clinically treated as acquisition failure and were clinically excluded from our datasets. In our datasets, only patient respiratory motion was observed. The motion mostly resulted in additional blurring rather than actual displacement of image structure. However, it is important to note that image alignment of breast volumes at different time frames may improve the accuracy of our analyses.

There are some limitations of this study, First, this was a retrospective analysis of images from a single vendor acquired at a single institution, although the analysis was conducted with independent training and testing sets with unique patients. It will be critical to evaluate whether the present findings generalize to other vendor images and external data. A future multicenter study may help address this question. Second, all the cases had gone to biopsy, thus, we could not assess the system on benign lesions that were deemed benign solely by follow-up. Also, the study findings cannot be used to determine whether the radiologists’ performances with the computer aid system are significantly improved in comparison with their performances without computer aid, even though we analyzed the DCE-MRI diagnostic results by the clinical radiologists. A clinical observer study is necessary. We note that we previously demonstrated in an observer study that use of computer-aided diagnosis with MRI improves the performance of radiologists in the task of differentiating malignant and benign lesions [29].

## Conclusions

In conclusion, we have validated a machine-learning radiomics method for DCE-MRI on an independent, consecutive patient test set, suggesting a potentially useful aid for radiologists in the task of distinguishing between malignant and benign breast lesions during diagnostic workup of breast lesions.

## Data Availability

NA

## Abbreviations

AI: Artificial intelligence
AUC: Area under the receiver operating characteristic curve
BI-RADS: Breast Imaging Reporting and Data System
CADx: Computer-aided diagnosis
CI: Confidence interval
DCE-MRI: Dynamic contrast-enhanced magnetic resonance imaging
DCIS: Ductal carcinoma in situ
IDC: Invasive ductal carcinoma
NPV: Negative predictive value
PPV: Positive predictive value
ROC: Receiver operating characteristic
SVM: Support-vector machine
